# Salicylate decreases the spontaneous firing rate of guinea pig auditory nerve fibres

**DOI:** 10.1016/j.neulet.2021.135705

**Published:** 2021-03-16

**Authors:** Mark N. Wallace, Christian J. Sumner, Joel I. Berger, Peter A. McNaughton, Alan R. Palmer

**Affiliations:** a Medical Research Council Institute of Hearing Research, School of Medicine, University of Nottingham, Nottingham, UK; b Hearing Sciences, Division of Clinical Neuroscience, School of Medicine, University of Nottingham, Nottingham, UK; c Wolfson Centre for Age-Related Disease, King's College London, London, UK

**Keywords:** CAP, compound action potential, CF, characteristic frequency, HSR, high spontaneous rate, LSR, low spontaneous rate, MSR, medium spontaneous rate, NMDA, N-methyl-d-aspartic acid, Vestibulocochlear nerve, Ototoxicity, Temporary tinnitus, Phantom limb sensation, Tinnitus mechanisms

## Abstract

Tinnitus has similarities to chronic neuropathic pain where there are changes in the firing rate of different types of afferent neurons. We postulated that one possible cause of tinnitus is a change in the distribution of spontaneous firing rates in at least one type of afferent auditory nerve fibre in anaesthetised guinea pigs. In control animals there was a bimodal distribution of spontaneous rates, but the position of the second mode was different depending upon whether the fibres responded best to high (> 4 kHz) or low (≤4 kHz) frequency tonal stimulation. The simplest and most reliable way of inducing tinnitus in experimental animals is to administer a high dose of sodium salicylate. The distribution of the spontaneous firing rates was different when salicylate (350 mg/kg) was administered, even when the sample was matched for the distribution of characteristic frequencies in the control population. The proportion of medium spontaneous rate fibres (MSR, 1≤ spikes/s ≤20) increased while the proportion of the highest, high spontaneous firing rate fibres (HSR, > 80 spikes/s) decreased following salicylate. The median rate fell from 64.7 spikes/s (control) to 35.4 spikes/s (salicylate); a highly significant change (Kruskal-Wallis test *p* < 0.001). When the changes were compared with various models of statistical probability, the most accurate model was one where most HSR fibres decreased their firing rate by 32 spikes/s. Thus, we have shown a reduction in the firing rate of HSR fibres that may be related to tinnitus.

## Highlights

• Spontaneous firing rates were recorded from single auditory fibres *in vivo*.
• Salicylate was injected at 350 mg/kg by the subcutaneous route.
• Median firing rate decreased by 32 spikes/s in nerve fibres after salicylate injection.
• The high spontaneous rate fibres (type 1A) showed the main reduction.

## Graphical abstract

**Figure:**
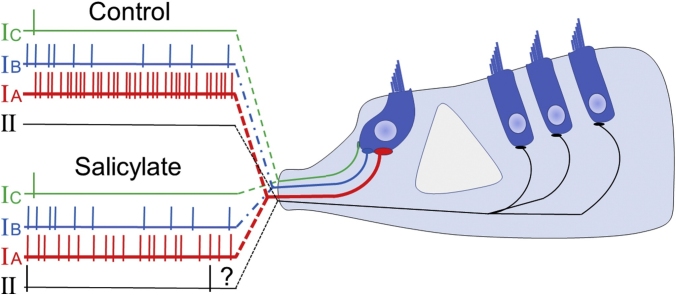

## 1 Introduction

Despite the prevalence of tinnitus in the general population, the mechanisms that produce this sometimes devastating sensation are still not satisfactorily understood and there is no broadly effective way to treat it [1]. Some researchers have emphasised the importance of central mechanisms involving the auditory cortex and limbic system [2], but the induction of tinnitus is usually associated with pathological activity in one or more cranial nerves – particularly the auditory nerve [3]. The degree to which altered peripheral activity is necessary to maintain tinnitus is still controversial, but is potentially important in the search for a pharmacological treatment.

There are four major types of afferent nerve fibre in the cochlear nerve that have been defined by their molecular profile [[4], [5], [6]] and their physiological profile [7,8]. These comprise three types of type I myelinated fibres, with spontaneous firing rates of between 0.01 and 140 spikes/s [8] that each innervate a single inner hair cell, and type II unmyelinated fibres that seem to show no spontaneous activity and each innervate multiple outer hair cells [9]. The type II fibres are thought to start firing in response to tissue damage [10] but they only form 5% of the overall population and their small size means we were unlikely to have recorded from even that many. The type I fibres in the guinea pig have been split into three groups on the basis of their spontaneous firing rate, with the largest group (HSR, 73%) having high rates of > 20 spikes/s, the medium group (MSR, 15%) having rates of 1≤ spikes/s ≤20 and the low group (LSR, 12%) having rates of <1 spikes/s [8]. Tinnitus is particularly noticeable in a silent background and in this study we wished to look for evidence that tinnitus is associated with altered levels of spontaneous activity in the auditory nerve that might be amenable to treatment with a peripherally acting drug.

One of the most reliable ways of inducing tinnitus, in an experimental setting, is to administer high doses of sodium salicylate (200−400 mg/kg) [11]. This model is based on clinical experience which showed that high doses of aspirin or its active breakdown product, salicylate, are associated with hearing loss and tinnitus [12]. At very high concentrations (equivalent to ≥400 mg/kg *in vitro*), salicylate activates the NMDA channels on nerve endings surrounding inner hair cells and increases the spontaneous firing rate of some afferents [13]. However, drugs that block peripheral NMDA receptors *in vivo* such as memantine and esketamine have not been successful in treating tinnitus clinically [1]. Different effects on spontaneous rate have been reported, depending on the concentration of salicylate. At near-lethal doses of 400 mg/kg *in vivo*, the mean spontaneous firing rate in the cat was found to increase [14], while at 200 mg/kg the mean rate in the cat showed no overall change [15] and the mean rate in the gerbil was found to decrease [16]. In this study we aimed to determine the effect of salicylate on auditory nerve firing rates using an intermediate level of salicylate (350 mg/kg), which we have previously used acutely to produce the sensation of tinnitus without any serious adverse effects on general health [17,18].

## 2 Materials and methods

### 2.1 Animals and surgical preparation

All procedures complied with the UK Animals (Scientific Procedures) Act 1986 and the EU Directive 2010/63/EU following approval by the Ethical Review Body at the University of Nottingham. Ten adult, tricolour guinea pigs, were split into two groups: a control group (4 ♀ & 1 ♂; weights 473–915 g) and a salicylate group (4 ♀ & 1 ♂; weights 489–742 g). They were anaesthetized with urethane (Sigma, 20% w/v at 4.5 mL/kg i.p. supplemented by 0.2 mL Hypnorm as described previously [19] to maintain areflexia, along with a single dose of atropine sulphate (s.c.) and 5 mL of warm, sterile saline i.p. to keep the animal hydrated. In the salicylate group, animals were given 350 mg/kg sodium salicylate dissolved at 200 mg/ml in distilled water s.c. at least 2 h before starting recordings from the auditory nerve. All animals were tracheotomized and and attached to an artificial respirator (100% O_2_; 100 cycles/min). A CO_2_ analyser was used to maintain end tidal CO_2_ (28–38 mm Hg). Core body temperature was maintained at 38 ± 0.5 °C. Animals were placed in a stereotaxic frame, with hollow earbars, inside a double-walled, sound-attenuating booth. A silver ball electrode was placed on the round window, the cerebellum was gently displaced with a spatula and the flocculus removed by aspiration, while being continually irrigated with warm (37 °C), sterile 0.9% saline [19].

### 2.2 Stimulation and recording

Aluminosilicate glass micropipettes (Clarkes SM100F-10, Harvard Apparatus Ltd., Edenbridge, UK) filled with 2.7 mKCl (starting impedances, 80–150 MΩ) were introduced into the left auditory nerve using a Burleigh Inchworm microdrive. The craniotomy was filled with 1.5% agar in 0.9% saline to prevent desiccation and to aid stability. For all analyses, the microelectrode signals (recorded with an Axon Instruments Axoprobe 1A) were amplified (×1000) and filtered (300–2000 Hz), and the time of occurrence of the spike was recorded with 1 μs accuracy (using TDT spike conditioner PC1, spike discriminator SD1, and event timer ET1; Tucker-Davis Technologies, Alachua, FL, USA).

Auditory stimuli were delivered monaurally via a closed-field system as described previously [19]. The maximum output level of the system was limited to approximately 100 dB SPL. The compound action potential (CAP) threshold to a 50 μs broadband click was assessed periodically. In the control animals, if the click CAP threshold was elevated by more than 10 dB then the experiment was terminated. Pure tones of duration 50 ms and rise–fall time of 2 ms presented every 200 ms were used to determine the characteristic frequency (CF) of units. Our search stimulus was mostly a broadband noise (0.02–16 kHz) at levels of up to approximately 80 dB SPL RMS but in low-frequency parts of the nerve pure tones were used. Spontaneous activity was collected for a minimum of 50 spikes and sometimes > 10 min. Spike size was constantly monitored to make sure the spikes were always above threshold. In addition to these data we also had access to data from 379 fibres recorded from the auditory nerve previously in the Palmer laboratory [[19], [20], [21]].

### 2.3 Statistical modelling of auditory nerve fibre populations

Spontaneous firing rate varies with CF in the rodent [22] and we sought to separate the effect of different sample populations from the effects of salicylate. Thus, we constructed statistical models of the control and salicylate data to quantify the differences between them. We modelled the distribution across CF with a smooth probability function, based on two-dimensional histograms (CF vs. spontaneous rate), smoothed by 2-dimensional convolution and normalised to create probability density functions (i.e. the area under the distribution summed to one). CF was divided into 9 log-spaced frequency groups from 0.2–51.2 kHz and smoothed with a triangular smoothing window (weights: 0.25,0.5,0.25). Spontaneous rate was divided into 16 bins (10 spikes per second steps, with bin ranges 0–10, 11−20, etc ….) and smoothed with a 5-term triangular smoothing window (0.125, 0.25, 0.5, 0.25, 0.125). The resulting function describes the probability of encountering a fibre with a given combination of CF and spontaneous rate.

The difference in the sampling of CF under salicylate was modelled by taking the marginal probability density function across CF (i.e. the overall probability of encountering a given CF irrespective of spontaneous rate) from the salicylate model, and computing a new probability density function where the distribution of spontaneous rate in each frequency bin was drawn from the control data. This represented the data which would be expected if the only changes were due to sampling of fibres with different CFs.

In a second model, we took the above model of CF change and additionally imposed a wholesale reduction in the spontaneous rate. This was done by resampling the probability functions in each CF group to model a reduction in firing rate by a fixed amount. For mathematical simplicity this was done numerically, by creating an imaginary set of data of 10,000 neurons from the probability distribution. Spontaneous rates were constrained so that they could not reduce to below zero. In a third model we instead modelled a proportionate reduction in firing rate across the population using the same resampling method.

In order to quantify the source of differences between the datasets, we compared how these different models could account for the salicylate data. The basic measure of model fit to the data is the likelihood of observing those data given the model. This is conveniently done by summing the log of the probability of observing each nerve fibre, *a_i_*, which has a known CF and spontaneous rate, given model *M*. The resulting *log-likelihood* function for dataset *A* (control or salicylate) is given by:

$$LL\left(A|M\right)=\sum_{i=1\;\ldots \;N}^{}ln\left[\;p\left(a_{i}\;|\;M\right)\;\right]$$

Log-likelihood values are *relative* and mean little in absolute terms. They are compared between different models applied to the same data using the Akaike Information Criterion (AIC), which is given by 2k-2 LL, where *k* is the number of parameters in the model. A smaller number indicates a better fit to the data, and thus models with larger numbers of parameters are penalised. It is additionally possible to compare models statistically if they only differ by a specific number of parameters using a likelihood ratio test. We applied this to the only applicable instance in our analysis: the difference between the model which accounts for the change in CF distribution-only and the model which adds a single parameter to modify spontaneous rate.

## 3 Results

### 3.1 Comparison of spontaneous firing rate in salicylate and control populations

**Fig. 1:**
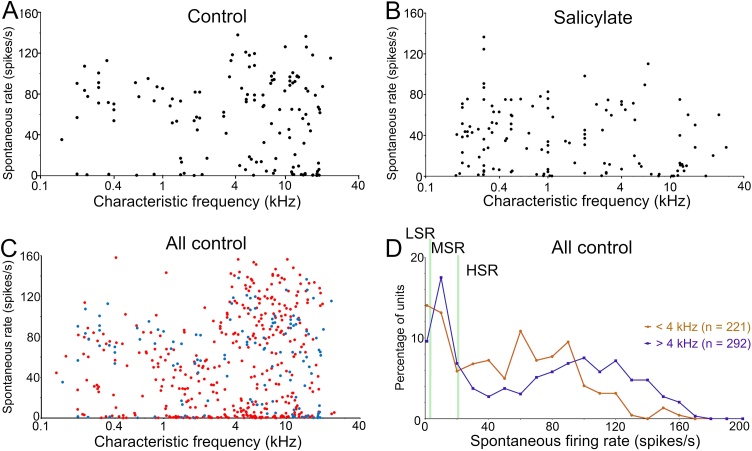
Scatter plots of CF versus spontaneous firing rate for the sample of fibres in the animals in the current control sample (A) and the salicylate sample (B). (C) Scatter plot of spontaneous firing rate against CF for 512 fibres from control groups of three previous studies (red dots; [[19], [20], [21]]) and the present one (blue dots). (D) Plot showing the percentage of units in a sequence of bins of mainly 10 spikes/s intervals for all 512 control fibres split into two groups based on their CF. The first two bins are different in that the first bin is for units with rates < 1 spikes/s and the second from 1 to 10 spikes/s. The pale green vertical lines mark the borders between the three subtypes of type I fibres.

Recordings were made from 122 fibres in animals where salicylate had been administered between 2 and 5 h previously. Previous data from our own group indicated behavioural changes consistent with the presence of tinnitus are observed within this time after administration [17,18]. The spontaneous firing rates ranged from 0 to 136 spikes/s with a median of 35 spikes/s. The CFs ranged from 0.18 to 28.7 kHz with a mean of 3.65 (±5.6) kHz. When the type I fibres were divided into the three sub-types based on spontaneous rate the proportions were as follows: LSR 8%; MSR, 30%; HSR, 62%. In the population from separate control animals, recordings were made from 178 fibres with spontaneous firing rates from 0 to 138 spikes/s and a median of 65 spikes/s. The CFs ranged from 0.15 to 23.4 kHz with a mean of 6.72 (±6.1) kHz. The proportions of fibres classified by spontaneous rate were: LSR (<1 spikes/s), 8%; MSR (1≤ spikes/s ≤20), 17%; HSR (>20 spikes/s), 75%. The data implies that there had been a large reduction in the spontaneous rate among the HSR fibres in the salicylate group and that some of them were now classified as MSR fibres. However, a direct comparison was complicated by the fact that there were more high CF fibres in the control sample than in the salicylate sample. This is illustrated in Fig. 1A,B where the spontaneous firing rate was plotted against the CF. Comparing the two populations, there are more HSR fibres with a moderately high firing rate of 20–50 spikes/s in the salicylate sample and more HSR fibres with a very high firing rate of above 80 spikes/s in the control population. However, if the spontaneous rate of guinea pig fibres varies with CF as it does in the gerbil [22] part of the difference in the spontaneous rates may be due to taking samples with different mean CFs. There are more high-frequency fibres (CF > 4 kHz) in the control population and this means that for a valid comparison, the samples need to have matched frequency distributions.

Another variable that could change between the two populations was the hearing thresholds. To assess the health of the cochlea during the experiment, the threshold of the click-evoked CAP was measured after the end of surgery and at 2 h after the first single fibre recording was made (control) or 2 h after salicylate had been administered. In the control animals, the mean CAP threshold at the start was -50.2 ± 5.2 (range -46 to -59) dB relative to maximum output and after 2 h it was -44.2 ± 6.75 (range -37 to -50) dB. This was a rise of 6 dB and was significant with a paired *t*-test (p = 0.014). In the salicylate animals the CAP threshold at the start was -52.8 ± 3.9 (range -46 to -55) dB and by 2 h after the salicylate it had risen to -36.2 ± 7.6 (range -24 to -43) dB. This was a rise of 16.6 dB and was significant with a paired *t*-test (p = 0.01). The rise in thresholds between the two groups was also significantly different (*t*-test, p = 0.03).

**Fig. 2:**
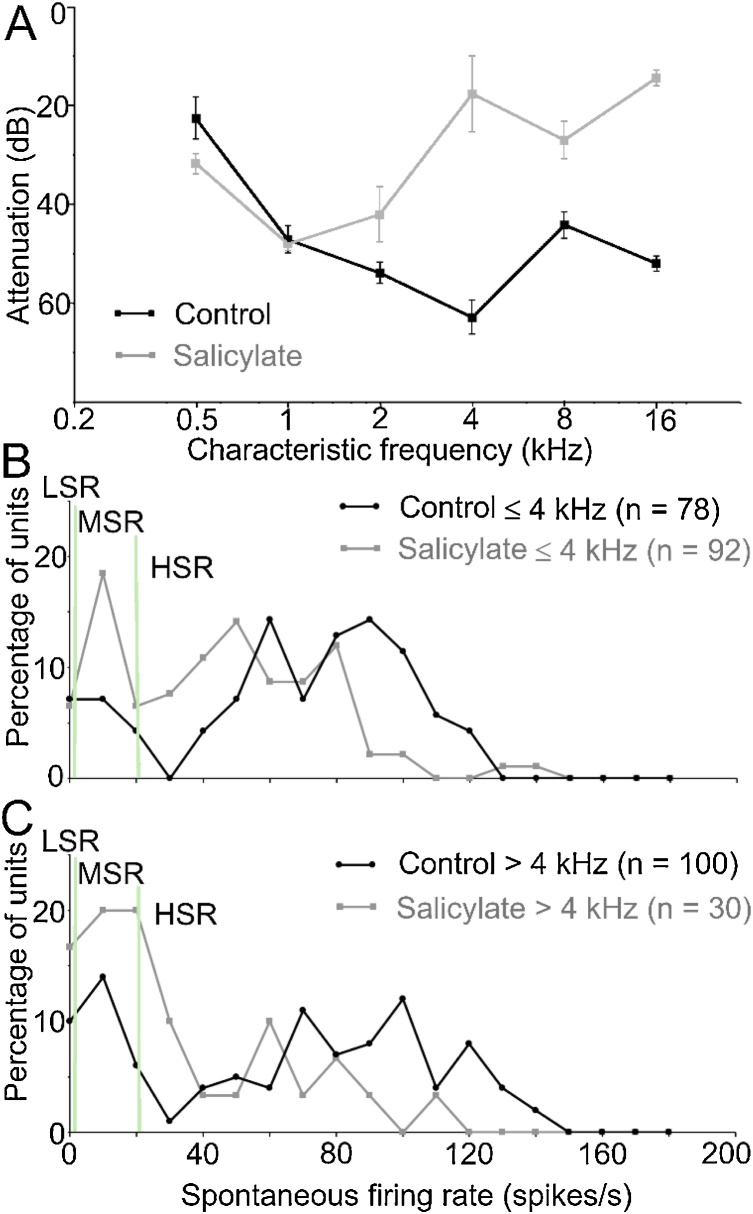
(A) Plots showing the mean threshold (± standard error) for the recorded units in the two populations using octave bands starting with all units with CFs ≤ 0.5 kHz. (B) Plots showing the percentage of units with a particular spontaneous rate in the same sequence of bins, as shown in Fig. 1D, for the two populations with low CFs. (C) Plots showing the same data for the populations with high CFs. In each case the pale green vertical lines mark the borders between the fibre subtypes.

The elevation in threshold likely varied with CF. To demonstrate this, mean thresholds for recorded units were calculated after binning units in octave bands, starting with those having CFs ≤ 0.5 kHz. This allowed us to compare mean thresholds across salicylate and control populations as shown in Fig. 2A. Salicylate caused a large increase in threshold of units with CFs of ≥ 2 kHz but there was no elevation of thresholds for neurons with CFs ≤ 1 kHz.

### 3.2 Dependence of spontaneous firing rate on characteristic frequency in the control population

To study the distribution of spontaneous firing rates among fibres with different CFs in a larger control sample we added the results from three of our previous auditory nerve studies. This gave a combined population of 512 units with spontaneous firing rates of 0–160 spikes/s and a median rate of 53 spikes/s. The CFs ranged from 0.07–24.7 kHz with a mean of 6.1 (±5.2) kHz. When the population was divided into fibre types based on spontaneous rate the proportion of different types was similar to that described previously [8]. The proportions of the different types were: LSR, 12%; MSR, 22%; HSR, 66%. When the CF was plotted against spontaneous rate (Fig. 1C) there was a break in the distribution, with fibres having CFs above about 4 kHz showing a greater probability of having a spontaneous firing rate of over 100 spikes/s. This was more clearly illustrated when the number of units (as a percentage) was plotted against the spontaneous firing rate of units grouped together in mainly 10 spikes/s intervals for low-frequency (≤ 4 kHz) and high-frequency (> 4 kHz) fibres (Fig. 1D). It showed that the two populations were different: there were more fibres with spontaneous rates of 30–90 spikes/s in the low CF group and more fibres with spontaneous rates of ≥ 100 spikes/s among the high CF group (confirmed with a Kruskal-Wallis test of spontaneous rates above and below 4 kHz CF, p < 0.001). This meant that when comparing the spontaneous rates of the control and salicylate populations it was important to compare samples with similar CFs.

The salicylate population and the control population from this study were each split into two groups based on having CFs ≤ 4 kHz or > 4 kHz to allow a comparison of spontaneous firing rates in groups that each had similar distributions of CFs. In the low-frequency comparison (Fig. 2B) there was a greater proportion of fibres in the range of 20–50 spikes/s for the salicylate group and a greater proportion of fibres in the range of 60–120 spikes/s in the control group. The two samples were compared with a Kruskal-Wallis test of similarity and found to be significantly different (p < 0.0001). A similar difference was found when comparing the fibres from the high CF groups (Fig. 2C). Again, the salicylate group showed a greater proportion of fibres with spontaneous rates in the range of 0–60 spikes/s while the control group showed a greater proportion of fibres in the range of 70–140 spikes/s. The two groups were also compared with a Kruskal-Wallis test and found to be significantly different (p = 0.0011). This confirms that there was a significant difference between the spontaneous firing rates of fibres in the control and salicylate populations, with a lower rate more common in the fibres of animals treated with salicylate.

### 3.3 Model to test the hypothesis that salicylate causes a general reduction in the firing rate of type I fibres

**Fig. 3:**
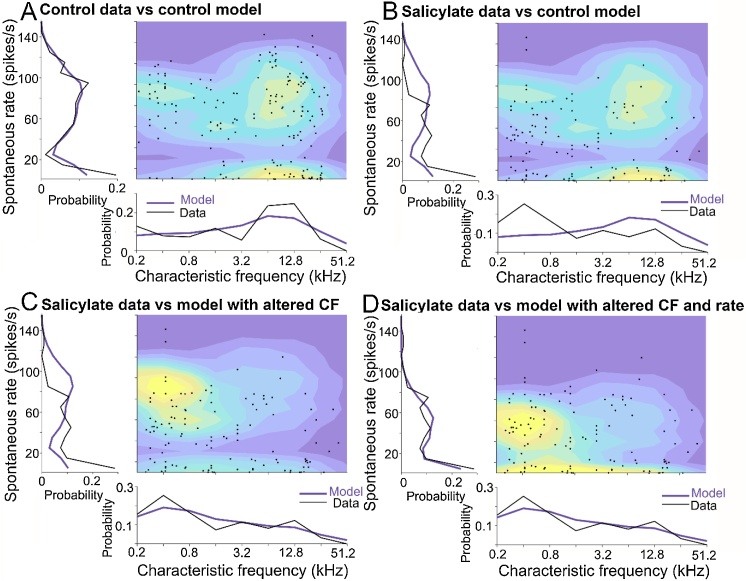
(A) Probabilistic model of spontaneous rate vs. CF for the control auditory nerve data shown as a contour map with warmer colours indicating a higher probability and the actual data superimposed as black dots. (B) Salicylate data (black dots) superimposed on the control contour map. (C) Contour map derived from the function for the control fibre rate probability and the salicylate CF sampling probability with the salicylate data superimposed. (D) A contour map based on the sampling CF function from the salicylate data and the spontaneous rate function from the control data after firing rate of all fibres has been reduced by 32 spikes/s unless this would make their value negative. This provided the value of best fit to the original salicylate data (black dots).

Although there was a clear effect of salicylate that was separate from any effect of mismatched sampling of CFs, it was useful to study the two parameters independently without making any assumptions about the position of the border between high and low CF fibres. We therefore produced simple probability models and then determined what steps would need to be implemented to transform the control data into the salicylate. By using the control data to calculate the probability of encountering a fibre with a particular spontaneous rate at a given CF (black lines in Fig. 3A) we can use smoothed probability functions (purple lines in Fig. 3A) to produce a contour map where hotter colours indicate a higher probability of recording from a fibre of that type. When the actual data is superimposed (black dots) there is a reasonably good fit with the data. However, when the salicylate data is superimposed on this same contour map (Fig. 3B) there is clearly a mismatch. Part of this is due to the sampling of fibres with different CFs and we can account for this by using a smoothed function based on the salicylate sampling to produce a new contour map with higher probabilities among the low CF fibres (Fig. 3C). This still does not produce a very good fit to the salicylate data until the spontaneous rate function is changed to reduce all the firing rates by 32 spikes/s, unless that would take them to a negative number. This increases the probability of recording from medium rate fibres and provides a much better fit to the data (Fig. 3D).

This analysis overall produces a family of models – probability maps predicting how likely it is to encounter a fibre with a given characteristic. We can compare these models against the salicylate data (Table 1) using the Akaike Information Criterion (AIC). A difference of >10 between AICs is considered as *strong* evidence that a model is better [23]. From the two models in Fig. 3C and D, we can additionally use a likelihood ratio test to quantitatively test whether the data reflect a change in spontaneous rate over and above what is expected from the sampling change. Formally, we test whether the model with the rate change is significantly more likely to give rise to the data than a model which only accounts for the difference in CF sampling. The result is very significant (χ^2^(1) = 88.2, *p* < .0001). Table 1 also shows the fit of a model where spontaneous rates were reduced proportionately (best fit was achieved with a reduction to 71% of the normal rates). The AIC provided strong evidence that this model did not account for the data as well as a fixed reduction.

**Table 1:** Comparison of models fitted to the salicylate data.

Model	AIC	AIC difference	χ^2^ statistic	p (χ^2^)
**Control data**	1167.5	–	–	–
**Control data + salicylate CF distribution**	1116.2	−51.2	–	–
**Control data + salicylate CF - 32 spikes/s**	1030.0	−86.2	88.2	<.0001
Control data + salicylate CF *71% **of normal rate**	1063.8	−52.4	–	–

## 4 Discussion

The main finding of this study was that in the presence of 350 mg/kg salicylate, injected s.c. between two and five hours previously, there was a large reduction in the median spontaneous firing rate of auditory nerve fibres compared with controls. This significant reduction in firing rate was present in fibres with both low CFs (≤ 4 kHz) and high CFs (> 4 kHz) and was mainly due to a big reduction in the numbers of fibres with spontaneous rates of above 80 spikes/s. It is consistent with the average spectrum of neural activity recorded from the round window of the guinea pig which showed a decrease in the mean spontaneous activity after an acute dose of salicylate [24]. In a study of single fibres in the gerbil there was also a reduction in the spontaneous firing rate following 200 mg/kg of salicylate, although the change was only significant among fibres with CFs < 5 kHz [16]. The spontaneous rate was measured when the basilar membrane was stationary and there would have been no input from the outer hair cells. Thus, this change in spontaneous rate should reflect a change in the properties of the synapses at the base of the inner hair cells. The changes in spontaneous rate occurred among fibres with both low and high CFs, unlike the changes in threshold which were only observed in fibres with CFs ≥ 2 kHz and not among fibres with CFs ≤ 1 kHz. This implies that the salicylate is potentially acting by two independent mechanisms: one involving synapses at the base of the inner hair cells [25], which is most obvious during silence and one involving the prestin motor and membrane stiffness of the outer hair cells that is involved in hearing sensitivity and dynamic responses to sound [26]. The lack of an effect of salicylate on thresholds among fibres with CFs ≤ 1 kHz may have two causes: 1) the fact that these low-frequency fibres already have higher thresholds than high-frequency fibres, in healthy controls [27] and 2) increased stiffness of the outer hair cell membrane caused by salicylate [28] may have a larger effect on high-frequency vibrations than on low ones.

To try to better understand the significance of our data to salicylate effects, we tried to fit several statistical probability models and found that the best model involved a constant reduction in spontaneous rate of 32 spikes/s in the HSR fibres for the salicylate condition. This has the effect of shifting control histograms (Fig. 2B,C) towards the left so that there are very few fibres with rates > 80 spikes/s and a larger peak at the lower end of the HSR fibres. As the model does not change the firing rate of any fibres with rates ≤ 20 spikes/s, they stay in place, but are added to by some fibres moving down from the HSR region. In practice this effect could be interpreted as salicylate having a specific effect on the synapses at the pillar side of the inner hair cells where the HSR (1A) fibres are located [29]. Salicylate apparently has little effect (at the concentration used in this study) on the modiolar side of the inner hair cell where the LSR and MSR synapses are located.

Relatively mild noise exposure, at a level and duration which does not produce a permanent hearing loss (106 dB SPL, 2 h), produces damage in the LSR and MSR fibres [30] but is insufficient to induce behavioural signs of tinnitus in the guinea pig [31] unless it is used on two occasions a few weeks apart when there may have been additional damage [32]. Two periods of noise exposure produced increases in spontaneous firing rate and synchrony among neurons of the dorsal cochlear nucleus that correlated with tinnitus [33]. Similarly, when salicylate was administered to guinea pigs in the same way as in this study, we previously showed that central plasticity can rapidly lead to increased activity in the auditory cortex that is correlated with tinnitus [18]. Thus, having a salicylate induced reduction in spontaneous firing rates of peripheral fibres is associated with the initiation of tinnitus [17] and suggests that altering activity in the peripheral nerve may be a viable target for early pharmaceutical intervention at least in forms of tinnitus originating in the cochlea [3].

## CRediT authorship contribution statement

**Mark N. Wallace:** Investigation, Formal analysis, Writing - original draft. **Christian J. Sumner:** Funding acquisition, Visualization, Software, Writing - review & editing. **Joel I. Berger:** Investigation. **Peter A. McNaughton:** Funding acquisition, Conceptualization. **Alan R. Palmer:** Conceptualization, Resources, Methodology, Supervision.

## Declaration of Competing Interest

The authors report no declarations of interest.
